# Isoproterenol infusion enhances composition and function of G-CSF mobilized allogeneic peripheral blood hematopoietic cell grafts

**DOI:** 10.1186/s13287-025-04725-4

**Published:** 2025-11-05

**Authors:** Helena Batatinha, Grace M. Niemiro, Nicole A. Peña, Giovannah A. Hoskin, Tiffany M. Zúñiga, Kyle A. Smith, Forrest L. Baker, Douglass M. Diak, Preteesh L. Mylabathula, Timothy M. Kistner, Michael D. Seckeler, Emmanuel Katsanis, Richard J. Simpson

**Affiliations:** 1 School of Nutritional Sciences and Wellness, The University of Arizona, Tucson, AZ USA; 2 Department of Pediatrics, The University of Arizona, Tucson, AZ USA; 3 The University of Arizona Cancer Center, Tucson, AZ USA; 4 Department of Immunobiology, The University of Arizona, Tucson, AZ USA

**Keywords:** Adrenergic receptors, Graft composition, AlloHCT, Leukemia.

## Abstract

**Background:**

Graft-versus-host disease (GvHD) and relapse remain critical challenges in allogeneic hematopoietic cell transplantation (alloHCT). Graft composition is pivotal, with naïve T cells increasing GvHD risk and NK cells improving graft-versus-leukemia (GvL) effects. Acute beta-adrenergic receptor activation mobilizes effector lymphocytes, favorably altering circulating immune cell composition. This study investigated whether infusing the non-selective beta-agonist isoproterenol (ISO) after granulocyte colony-stimulating factor (G-CSF) mobilization enhances peripheral blood hematopoietic cell (PBHC) graft composition and outcomes.

**Methods:**

Ten healthy volunteers received a 20-minute ISO infusion before and after five days of G-CSF hematopoietic cell mobilization. G-CSF and G-CSF + ISO mobilized PBHCs were phenotyped and assessed for in vitro cytotoxicity. NSG leukemia-bearing mice were injected with G-CSF or G-CSF + ISO mobilized PBHCs and monitored for GvHD, tumor burden, and overall survival.

**Results:**

After G-CSF mobilization, ISO increased the numbers of CD34 + cells in the blood and favorably altered graft composition, increasing NK (9.5% to 27.9%) and TCR-γδ T cells (5.0% to 7.5%) while reducing naïve CD4 (18.1% to 11.2%) and CD8 (8.9% to 5.8%) T cells. Effector lymphocytes mobilized by G-CSF + ISO, particularly effector-memory CD8 + T-cells and NK-cells, exhibited upregulated genes and enriched gene sets linked to anti-tumor activity (e.g. NKG7, GZMB, NK cells cytotoxicity). This resulted in an 8-fold increase in cytolysis against the K562 leukemia cell line compared to PBHC mobilized by G-CSF only. In xenogeneic mice, G-CSF + ISO grafts reduced GvHD, extended survival, and improved GvL effects, with 42% of mice surviving at day 40 compared to 21% for G-CSF grafts.

**Conclusions:**

ISO infusion post-G-CSF mobilization favorably enhances graft composition, mitigates GvHD, prolongs survival, and augments GvL effects. Our findings suggest that acute systemic beta-adrenergic receptor activation could be a valuable strategy to enhance outcomes in alloHCT.

**Graphical Abstract:**

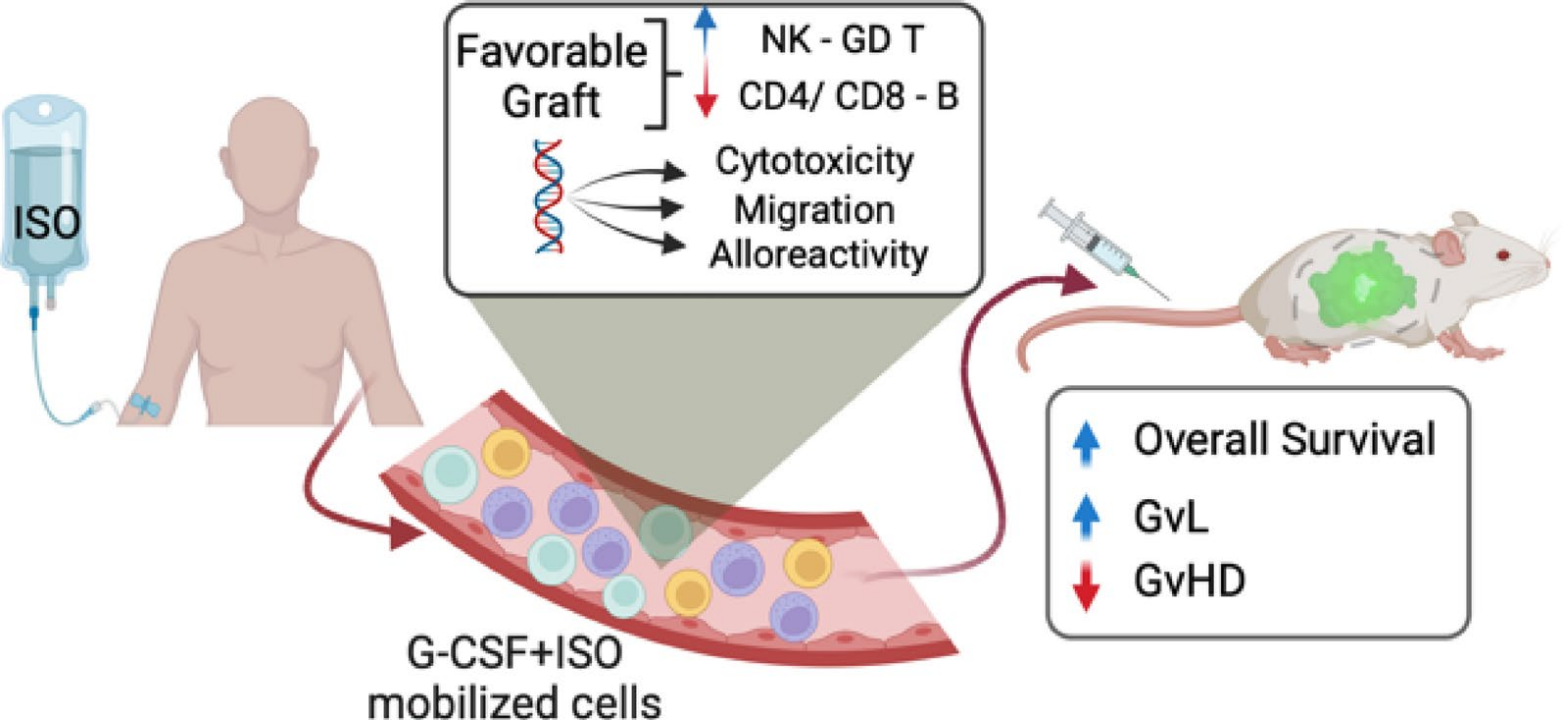

**Supplementary Information:**

The online version contains supplementary material available at 10.1186/s13287-025-04725-4.

## Introduction

Allogeneic hematopoietic cell transplantation (alloHCT) remains a curative option for many patients with hematologic malignancies, non-malignant blood disorders, primary immunodeficiencies, and other conditions [1, 2] Despite its potential, alloHCT is still associated with significant morbidity and mortality, particularly due to graft-versus-host disease (GvHD) [3, 4]. This risk is heightened when granulocyte colony-stimulating factor (G-CSF) mobilized peripheral blood hematopoietic cells (PBHCs) are used in place of bone marrow [4, 5]. High-dose corticosteroids and immunosuppressants are the first-line therapies for GvHD. Yet, their high failure rate—approximately 40%—and poor treatment outcomes remain significant challenges [6].

Numerous studies have explored depleting various cell types in the graft to prevent GvHD, but while these approaches have successfully reduced GvHD, they have also diminished the curative graft-versus-leukemia (GvL) effect – see [7] for an in-depth review. A study analyzing over 7,000 patients who underwent alloHSCT for different types of leukemia found that chronic GvHD had a protective effect against late relapse [8], underscoring the strong relationship between GvL and GvHD. These challenges emphasise the need for novel strategies to enhance the safety and efficacy of G-CSF-mobilized PBHC grafts.

Prospective studies using PBHCs for alloHCT have identified a “favorable” graft immunophenotype that mitigates GvHD while preserving GvL effects and promoting engraftment [9]. This favorable immunophenotype is characterized by an increased presence of CD34 + hematopoietic stem cells (HSCs), CD56dim natural killer (NK) cells, TCR-γδ cells, and memory CD8 + TCR-αβ cells, alongside reduced levels of less favorable cell types such as naive TCR-αβ (TN) cells, dendritic cells (DCs), and CD19 + B-cells [2, 10]. The enrichment of NK and TCR-γδ cells enhances antiviral defense and anti-tumor activity without triggering GvHD, while the reduction in TCR-αβ (especially TN) cells and B-cells helps prevent GvHD [11]. Although ex vivo depletion of TCR-αβ cells and CD19 + B-cells has been widely employed to reduce GvHD [12, 13], this method is labor-intensive and frequently associated with graft failure, delayed immune reconstitution, and a increased risk of infections and relapse [9, 14].

There is an unmet need to optimize the composition and function of G-CSF mobilized donor PBHC grafts, reducing reliance on suboptimal and labor-intensive ex vivo graft manipulation strategies. Our research has demonstrated that a single bout of moderate to vigorous intensity exercise mobilizes CD34 + HSCs and significantly alters the immune cell composition of peripheral blood, increasing the numbers and proportions of favorable cell types, such as NK and TCR-γδ cells, while reducing those of unfavorable types, including TN cells and B-cells [15, 16]. Furthermore, administration of these exercise-mobilized lymphocytes as donor lymphocyte infusions (DLI) in leukemia-bearing xenogeneic mice prolonged survival and reduced leukemic burden compared to standard DLI from the same donors [15]. We have also identified that the mobilization of CD34 + HSCs and changes in immune cell composition with exercise are mediated by the catecholamine- β2-adrenergic receptor (β2-AR) signaling pathway [16], suggesting that similar shifts in graft composition could potentially be achieved through pharmacological intervention rather than exercise. This approach could have significant clinical implications, as PBHC collections typically last 3–4 h, making it impractical to require donors to exercise for such prolonged periods. Moreover, exercise and β2-AR activation alone is insufficient to increase CD34 + HSC numbers to clinically relevant levels in healthy donors [16, 17], suggesting that this approach may only be effective following G-CSF mobilization.

In this study, we administered the synthetic β-AR agonist isoproterenol (ISO) to healthy donors before and after G-CSF mobilization. ISO significantly increased the circulating numbers of CD34 + cells following 5 days of G-CSF administration and promoted a favorable graft phenotype by increasing NK and TCR-γδ cells while reducing TN cells and B-cells. The G-CSF + ISO mobilized cells demonstrated enhanced cytotoxicity against the K562 leukemia cell line in vitro. Injection of G-CSF + ISO mobilized cells into xenogeneic mice, resulted in reduced GvHD and lower leukemia burden compared to standard G-CSF mobilized cells. These findings suggest that targeting the β2-AR could be a viable strategy to augment CD34 + cell yields and favorably modify the immune composition of G-CSF mobilized PBHC grafts, ultimately improving GvL effects while minimizing GvHD after alloHCT.

## Methods

### Participants

Ten healthy adults, (4 females, 6 males) participated in this study (Table 1). Written informed consent and medical history were obtained from each participant after a proper explanation of the procedures and risks. Only participants classified as ‘low risk’ for cardiovascular disease in accordance with the risk stratification guidelines published by the American Heart Association and the American College of Sports Medicine (AHA/ACSM criteria) were enrolled [12]. The study was approved by the Human Subjects Protection Program at the University of Arizona (#1711017841).

**Table 1:** Demographic data

Subject ID	age	Sex	Height (cm)	Weight (kg)	BMI	HR (Rest)	SBP (Rest)	DBP (Rest)
ISO.001	31	M	167	59.6	21.37	65	119	58
ISO.002	39	M	181	73.55	22.45	53	126	71
ISO.003	26	M	186	81.9	23.67	66	132	77
ISO.004	26	F	157.5	49.75	20.18	87	110	77
ISO.005	25	F	154	55.2	23.28	55	105	68
ISO.006	21	F	162.5	70.3	26.79	77	131	63
ISO.007	25	F	161.6	57.8	22.30	62	118	82
ISO.008	24	M	188	82	23.20	62	125	59
ISO.009	27	M	182.3	79.4	23.97	55	138	57
ISO.010	22	M	170.8	59.9	20.73	62	117	62
Average	**26.60**		**171.07**	**66.94**	**22.79**	**64.40**	**122.10**	**67.40**
SD	**5.15**		**12.43**	**11.93**	**1.88**	**10.52**	**10.25**	**8.98**

### Isoproterenol infusion, G-CSF mobilization, and blood sampling

Participants visited the laboratory on 7 separate occasions across 7 consecutive days. All laboratory visits occurred between 8:00 and 10:00. Participants were instructed to arrive at the lab following an 8–12 h overnight fast and to refrain from engaging in vigorous physical activity/exercise throughout the study period. **Visit 1**: Informed consent was obtained, and participants completed the AHA/ACSM pre-screening questionnaire. After the eligibility criteria were met, an intravenous catheter was inserted into each arm of the participant. One catheter was used to deliver isoproterenol (ISO) and the other to collect blood. Before ISO infusion, electrodes were placed on appropriate chest regions to collect electrocardiogram data (12-lead ECG) throughout the entire infusion period. Resting blood pressure and heart rate were recorded at this time. Each participant was then infused with ISO at a concentration of 50ng/kg/min for 20 min. The blood samples were collected before and during the last 5 min of ISO infusion. **Visits 2–6**: Participants were scheduled to visit the laboratory between 08:00 and 10:00 on 5 consecutive days. During each visit, they received a single 5mcg/kg/day subcutaneous dose of recombinant G-CSF (Filgrastim). The total dose of filgrastim received over the 5 day period was 25mcg/kg. **Visit 7**: The day after the last G-CSF injection, participants returned to the lab for the last visit, and followed the exact same procedures described in Visit 1. That is, a 50mL blood sample was collected at rest and during the last 5 min of a 20-minute ISO infusion.

### Blood processing and analysis

Blood samples collected in EDTA tubes underwent complete blood counts using an automated hematology analyzer (Beckman Coulter, Indianapolis, IN). To detect and quantify leukocyte and progenitor cell subpopulations, whole blood samples from EDTA tubes were labeled with monoclonal antibodies and analyzed using flow cytometry (MACSQuant 10, Miltenyi Biotec, San Diego, CA). Immune cell phenotypes were determined within the PBHC gate based on forward and side scatter using FlowLogic software (Inivai Technologies, Mentone Victoria, Australia). Electronic color compensation was achieved using single-color tubes and calibration beads, and Fluorescence Minus One (FMOs) controls were used to establish proper gating. A minimum of 50,000 PBHCs were collected for analysis. Details of the flow cytometry antibody panels used are provided in Supplementary Table 1. PBHCs were isolated from blood collected in ACD tubes and cryopreserved in liquid nitrogen at a concentration of 10 × 10^6^ cells/mL freezing media (90% FBS, 10% DMSO) until further analysis.

### Single-cell RNA sequencing

Thawed cryopreserved PBHCs from 5 donors were sent to the University of Arizona Genetics Core for single-cell RNA sequencing (scRNAseq) analysis utilizing the 10x Genomics platform. To prepare the 5’ RNA whole transcriptome libraries, the “10xGenomics Chromium Next GEM Single Cell 5’ reagents kit v2” was employed according to the manufacturer’s guidelines. After library generation, quantification, normalization, and pooling, sequencing was performed on an Illumina NextSeq500 sequencer. The FastQ files obtained from sequencing were processed into expression matrices using the “cellranger count” function provided by Cell Ranger (version 6.0.1). Unfiltered matrices were then imported into R (version 4.1.0). Empty droplets were identified and eliminated using the emptyDrops function from the DropletUtils package, while reads with a high mitochondrial content were filtered out using the perCellQCMetrics function provided by Scuttle. For downstream analysis, gene expression on a per-cell basis was assessed through principal component analysis (PCA) and uniform manifold approximation and projection (UMAP) clustering using Seurat (version 4.0.5). Differentially expressed genes were identified using the FindMarkers function in Seurat, with a log2 fold cutoff of 0. To gain insights into biological pathways and processes, gene set enrichment analysis (GSEA) was performed with FDR < 0.05. The results were then annotated to Kyoto Encyclopedia of Genes and Genomes (KEGG) and Gene Ontology (GO) terms.

### In vitro cytotoxicity

Thawed cryopreserved PBHCs were resuspended in media (RPMI supplemented with 10% FBS), and incubated for one-hour with IL-15 (0.1 mg/mL), to enhance activation and recovery of NK cell functions. Following incubation, PBHCs were washed and resuspended in media. Luciferase-tagged K562 (K562-luc2) were co-cultured with PBHCs at an E: T ratio of 0:1 (spontaneous death control), 1:1, 2.5:1, 5:1, and 10:1 for a total of 24 h in U-bottom 96-well-plates. Cytotoxicity was determined at four-hours and 24-hours by analyzing the changes in target cell bioluminescence following procedures we have described previously [18].

### Xenogeneic mouse experiments

The experiments described herein were approved by the University of Arizona Institutional Animal Care and Use Committee (IACUC) under protocol number 17–338. Eight- to twelve-week-old NSG mice (Jackson Labs, Stock No: 005557) were used for GvHD and human immune cell engraftment experiments. Prior to cell injection, all mice received a radiation dose of 100 cGy on Day − 2 using a Bio5 Cesium 137 Irradiator (Atomic Energy of Canada Ltd) to enhance the engraftment of human cells. On Day − 1, mice were intravenously injected with 5 × 10^6^ PBHCs obtained from either G-CSF or G-CSF + ISO samples. For the xenotransplantation experiments involving K562-luc2 tumor cells (ATCC, Manassas, VA) and human PBHCs, we utilized 8-12-week-old NOD.Cg-Prkdcscid Il2rgtm1Wjl Tg(IL15)1Sz/SzJ (NSG-Tg(Hu-IL15)) mice obtained from Jackson Laboratories (Stock No: 030890). These mice have a human IL-15 transgene, facilitating the expression of physiological levels of human IL-15 (7.1 ± 0.3 pg/mL). Mice were irradiated on Day − 2 (100 cGy). On Day − 1, mice were intravenously injected with 5 × 10^6^ PBHCs obtained from either G-CSF or G-CSF + ISO samples. On Day 0, mice were injected via the lateral tail vein with 5 × 10^5^ K562-luc2 cells. Bioluminescent imaging (BLI) commenced on day + 1 post-tumor injection and was repeated every 3–4 days to monitor tumor progression, utilizing the LargoX bioluminescence imager (Spectral Instruments Imaging, Tucson, AZ). The three experimental groups were: K562.Vehicle, K562.G-CSF, K562.G-CSF + ISO. Mice not receiving tumor or PBHC injections were instead administered an equal volume of saline. Throughout the experimental period, all animals were monitored daily until the time of sacrifice. PBHC samples were injected in duplicate or triplicate, with 2–3 mice per human PBHC sample. All mice were euthanized using a lethal dose of CO2 at day + 40. For the assessment of GvHD, the experiment was repeated 5 times to achieve 10 mice per group (total of 20 mice). To determine GvL effects, the experiment was repeated 4 times to achieve 14 mice in each experimental group and 5 mice in the control group (total of 33 mice). Group allocation was performed by an individual who was not involved in data collection or analysis. The person responsible for data collection and analysis was blinded to the group assignments.

### Leukemia cells and PBHC Preparation for injection

K562-luc2 cells were thawed from cryopreservation 48 h before tumor injections and cultured at 37 °C with 5% CO2 in Iscove’s DMEM supplemented with 10% FBS and 8 µg/mL blasticidin for 2 days. On the day of tumor injections, K562-luc2 cells were harvested from culture, washed three times with sterile PBS, and then suspended in sterile saline at a concentration of 5 × 10^5^ cells per 200µL. Cryopreserved PBHCs were thawed in a 37 °C water bath for two minutes until a small ice ball remained in the solution. Subsequently, PBHCs were resuspended in 5mL RPMI supplemented with 10% FBS, supplemented with IL-15 (0.1 mg/mL), and incubated for 1 h at 37 °C with 5% CO2 to enhance activation and recovery of NK cell functions. Following incubation, PBHCs were washed three times with PBS to remove all RPMI media and then resuspended at a concentration of 5 × 10^6^ PBHCs per 200µL sterile filtered saline for injections.

### Immune cell engraftment

Human immune cell engraftment was monitored on a weekly basis. Blood samples (50µL) were collected from each mouse using the submental method. For flow cytometric analysis, 25µL of whole blood samples were stained with directly conjugated antibodies, including CD8-VioBlue, CD14-VioGreen, CD3-FITC, CD4-PE, CD20-PerCP, CD45mouse-PE-Vio770, CD45human-APC, and CD56-APC-Vio770 (all from Miltenyi Biotec). All antibodies, with the exception of CD45mouse, targeted human antigens. CD45mouse was specifically utilized to exclude mouse leukocytes from the analysis.

### Bioluminescence imaging and GvHD scoring

All mice underwent bioluminescent (BLI) imaging every 3–4 days to track tumor progression using the LargoX bioluminescent imager. To initiate imaging, mice were intraperitoneally injected with D-luciferin, potassium salt reconstituted in Dulbecco’s phosphate-buffered saline (15 mg/ml) (Gold Biotechnologies, St. Louis, MO) at a concentration of 10µL/g body weight (BW). Bioluminescent images were captured using various exposure times ranging from 5 min to 1 s, and the bioluminescent data was quantified as photons per second (photons/s). On the same day as imaging, mice were evaluated for symptoms of xenogeneic GvHD and weighed. GvHD symptoms were scored based on clinical observations, including skin and fur integrity, posture, activity level, weight loss, and the presence of diarrhea [15]. Each symptom was graded on a scale from 0 to 11, with higher scores indicating more severe symptoms. Mice exhibiting multiple combinations of clinical signs indicative of a moribund condition were euthanized accordingly. Due to the inability to measure actual tumor volume following intravenous administration of K562 cells, euthanasia criteria included GvHD severity reaching or exceeding 8, body weight loss of 20% or more, hind limb paralysis, impaired ambulation, or any other signs of distress.

### Statistical analysis

To identify significant differences in the total numbers of immune cell subtypes measured in blood across all four time points, we utilized One-Way ANOVA. For assessing the main effects of time, group, and their interaction (time x group) on human leukocyte engraftment, GvHD, and leukemic burden measured by BLI (photons/s), we employed linear mixed models. Bonferroni correction was applied for multiple comparisons. To analyze survival outcomes, including overall survival, tumor-free survival, and GvHD-free survival, we conducted Kaplan-Meier survival analysis. We also compared days to death and days to tumor growth between groups using one-way ANOVA (3-groups) and independent t-tests (2 groups). Furthermore, the percentage of surviving mice with low leukemic burden (defined as a BLI score below the 95% confidence interval of control mice receiving only K562 cells) between groups on a weekly basis using chi-square tests. Statistical significance was determined at a threshold of *p* < 0.05. For differential gene expression (DGE) analysis based on single-cell RNA sequencing (scRNAseq), we considered fold changes in individual gene expression within each cluster significant after adjusting for multiple hypothesis testing (Padj < 0.05). In gene set enrichment analysis (GSEA), false discovery rate (FDR) correction was applied separately for each database (Gene Ontology and Kyoto Encyclopedia of Genes and Genomes) to correct for multiple hypothesis testing, with significance defined as FDR < 0.05.

## Results

### ISO infusion post-G-CSF mobilization favorably alters peripheral blood cell composition

**Fig. 1:**
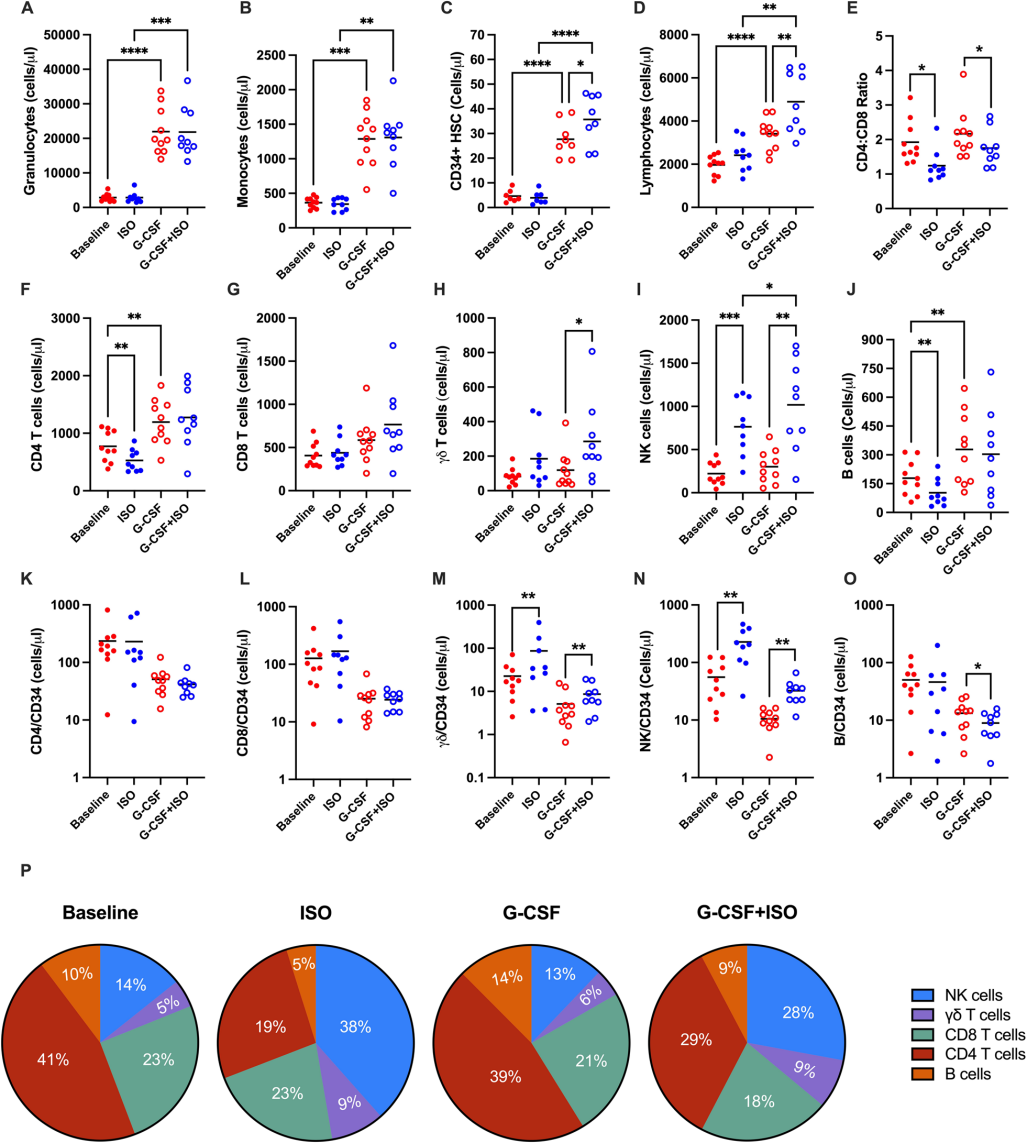
ISO Infusion Modifies the Composition of G-CSF Mobilized PBHCs. Total cell count (cells/µL) of major leukocyte populations (**A–D**) and lymphocyte subpopulations (**F–J**) were measured at baseline (solid red), after ISO infusion (solid blue), after G-CSF mobilization (open red), and after combined G-CSF + ISO mobilization (open blue). (**E**) CD4:CD8 ratio. Total cell count (cells/µL) of (**K**) CD4⁺ T cells, (**L**) CD8⁺ T cells, (**M**) γδ T cells, (**N**) NK cells, and (**O**) B cells were normalized to CD34⁺ HSC counts. (**P**) Proportional representation of lymphocyte populations in grafts collected under each condition (baseline, ISO, G-CSF, and G-CSF + ISO). Data are presented as mean ± SEM; *N* = 10. Statistical significance: *P ≤ 0.05, **P ≤ 0.01, ***P ≤ 0.001, ****P ≤ 0.0001

Ten healthy volunteers received isoproterenol (ISO) infusions both before and after a 5-day course of G-CSF. Samples were collected at four time points: baseline, after ISO infusion (ISO), at rest following G-CSF mobilization (G-CSF), and during ISO infusion after the G-CSF mobilization (G-CSF + ISO). One subject did not complete the G-CSF + ISO blood draw. Systolic blood pressure and heart rate were elevated during ISO infusion, with no significant difference between pre- and post-G-CSF mobilization (Supplementary Fig. 1). G-CSF elicited a significant mobilization of granulocytes, monocytes, and hematopoietic stem cells (HSCs) (Fig. 1A, B, D), consistent with expectations. Notably, there was also an increase in total lymphocytes, particularly in CD4 + T cells and B cells (Fig. 1C, F, J). The infusion of ISO had an additional effect, significantly increasing the total count of G-CSF-mobilized CD34 + cells and lymphocytes. Among the lymphocytes, NK cells and γδ T cells were the predominant contributors (Fig. 1H, I). Although no statistical differences were observed in the CD8 + T cells, the CD4:CD8 ratio decreased following ISO infusion (Fig. 1E).

**Fig. 2:**
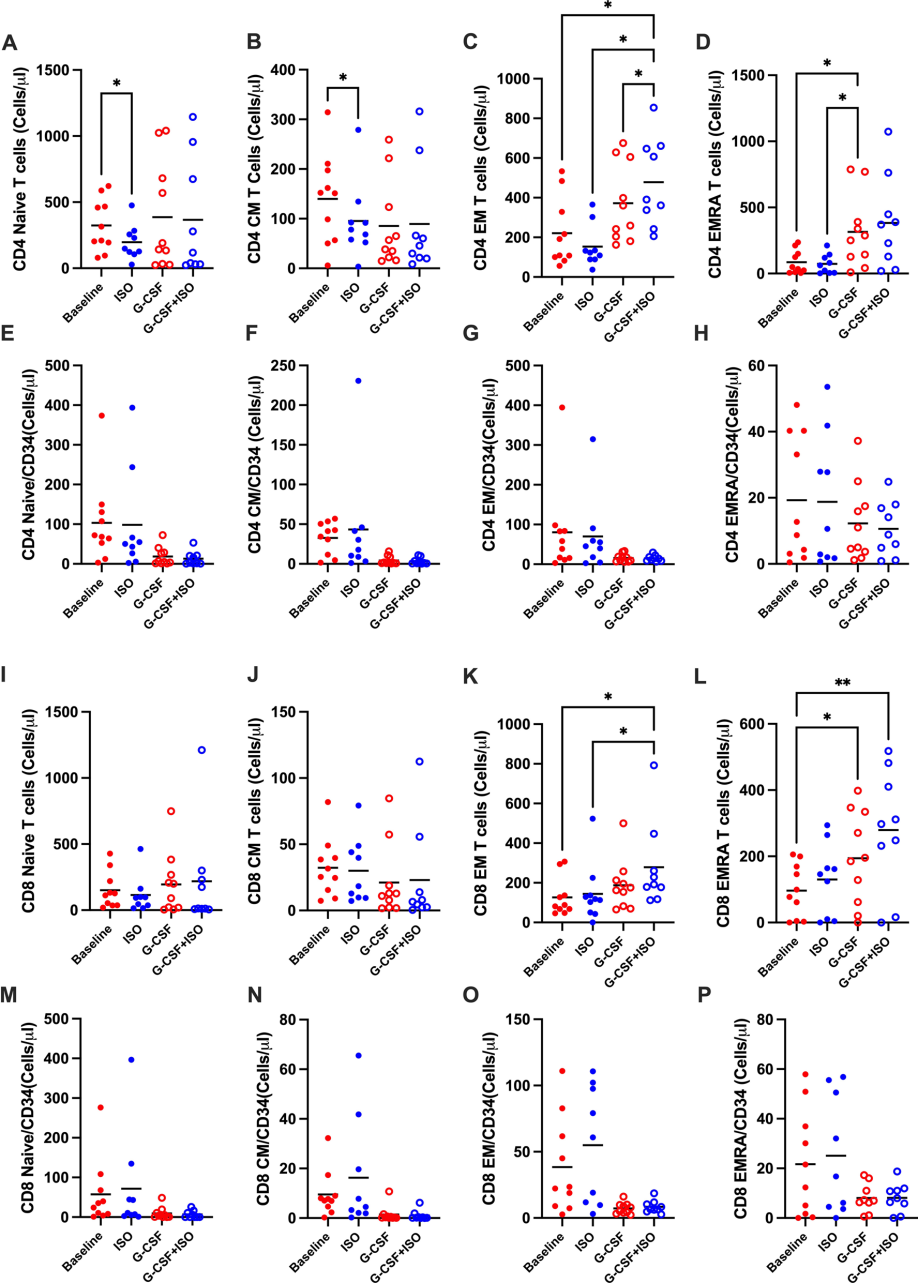
ISO Infusion Following G-CSF Mobilization Enhances Effector Memory Lymphocyte CompositionTotal cell count (cells/µL) of CD4⁺ T cell memory subtypes (A–D) and their counts normalized to CD34⁺ HSC numbers (E–H). Total cell count (cells/µL) of CD8⁺ T cell memory subtypes (I–L) and their normalized counts (M–P). Cells were collected at baseline (solid red), after ISO infusion (solid blue), after G-CSF mobilization (open red), and after combined G-CSF+ISO mobilization (open blue). Data are presented as mean ± SEM; N = 10. Statistical significance: *P ≤ 0.05, **P ≤ 0.01

Given that CD34 + cell counts regulate the cellular composition of HSC grafts during transplantation., a breakdown of cell types per CD34 + cell count was conducted. Administration of ISO after G-CSF mobilization was found to increase the number of favorable cells—likely to induce graft-versus-leukemia (GvL) effects, such as NK and γδ T cells—while decreasing the number of unfavorable cells—likely to provoke GvHD, such as B cells—per CD34 + cell count (Fig. 1K-O). With respect to differentiated T cell subsets, the combined administration of G-CSF and ISO increased effector memory (EM) CD4+ (Fig. 2C) and CD8 + T cells (Fig. 2K), as well as terminally differentiated effector memory (EMRA) CD8 + T cells (Fig. 2L). However, this effect was diminished when normalized by CD34 + cell counts (Fig. 2E-H, M-P).

Furthermore, ISO infusion improved the composition of G-CSF-mobilized PBHCs, by increasing the proportion of NK cells and γδ T cells while decreasing les favorable components such as B cells, and CD4 + T cells (Fig. 1P). This shift suggests a potential enhancement of the GvL response with reduced GvHD risk.

### Single-cell RNA sequencing reveals enrichment of gene sets associated with anti-tumor activity in G-CSF + ISO mobilized PBHCs

**Fig. 3:**
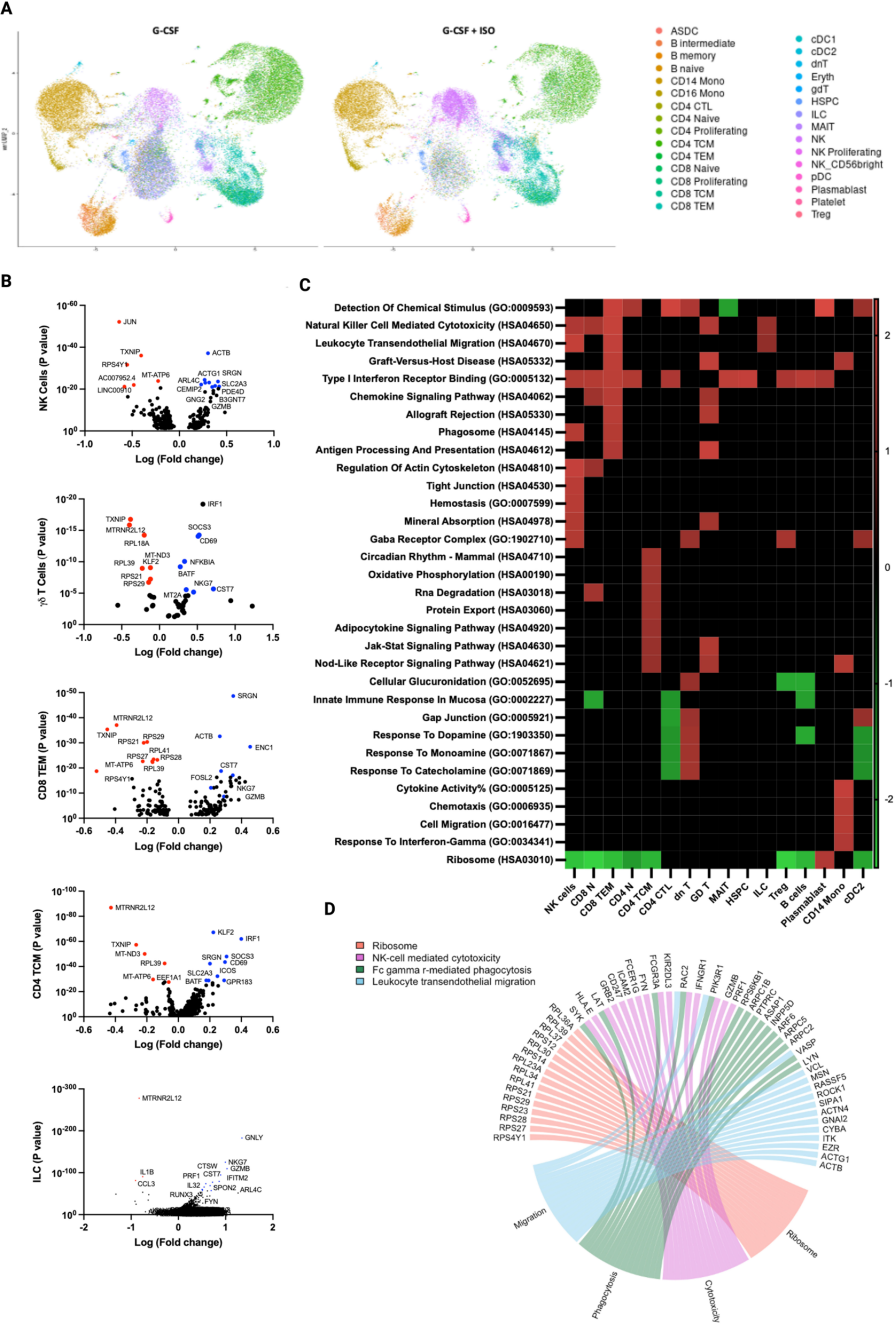
-CSF + ISO Mobilized PBHCs Exhibit Gene Expression Profiles Linked to Enhanced Anti-Tumor Immunity. Single-cell RNA sequencing was performed to identify genes and pathways that are differentially regulated in G-CSF + ISO mobilized grafts. (**A**)Azimuth map of human PBHCs mobilized by G-CSF alone or G-CSF + ISO, highlighting 30 distinct clusters identified via scRNA-seq. (**B**) Volcano plot representing differentially expressed genes between G-CSF and G-CSF + ISO across five immune cell subsets. (**C**) Gene sets associated with GO and KEGG pathways enriched following G-CSF + ISO mobilization. (**D**) Chord diagram displaying the leading-edge genes driving the enrichment of terms associated with cytotoxicity and anti-tumor signaling in NK cells. All genes and pathways presented in this figure are statistically significant (*P* ≤ 0.05)

To investigate the effects of ISO infusion on the transcriptome within G-CSF mobilized PBHCs, we performed scRNAseq and identified 30 cell clusters following the Azimuth map for human PBHCs (Fig. 3A). Differentially Expressed Genes (DEGs) between G-CSF and G-CSF + ISO mobilized PBHCs were observed in 7 cell types. Genes associated with increased cytotoxicity (*GZMB*,* GNLY*,* PRF1*), and anti-tumor activity (*NKG7*) were upregulated in the cytotoxic lymphocytes, including CD8 + T cells, γδ T cells, NK cells, and ILCs. CD4 + T cells exhibited enrichment of genes associated with T cell differentiation and IFN-γ production (*KLF2*,* ICOS*,* IRF1*). Ribosomal and mitochondrial genes (*RPLs*,* MTs*) were downregulated across all seven cell types (Fig. 3B).

To identify biological processes associated with our DEGs data, we performed functional annotation and enrichment analysis using both GO and KEGG terms. We observed an upregulation of enriched gene sets associated with cytotoxicity, cellular migration, and anti-tumor activity within NK cells, CD8 + T cells, γδ T cells, and ILCs. Type I interferon receptor binding was upregulated in most cell types, including HSPCs, cytotoxic lymphocytes, B cells, Tregs, and Monocytes. Gene sets involved in oxidative metabolism were enriched in CM CD4 + T cells. Interestingly, gene sets involved in protein synthesis (e.g., ‘ribosome’ and ‘translation’) were downregulated in most cell types in response to ISO infusion (Fig. 3C). These findings indicate that ISO infusion significantly alters the transcriptome of G-CSF mobilized PBHCs, particularly enhancing the expression of genes associated with cytotoxicity and anti-tumor activity in various lymphocyte subsets.

### ISO + GCSF mobilized grafts mitigate GvHD and prolong survival in xenogeneic mice

**Fig. 4:**
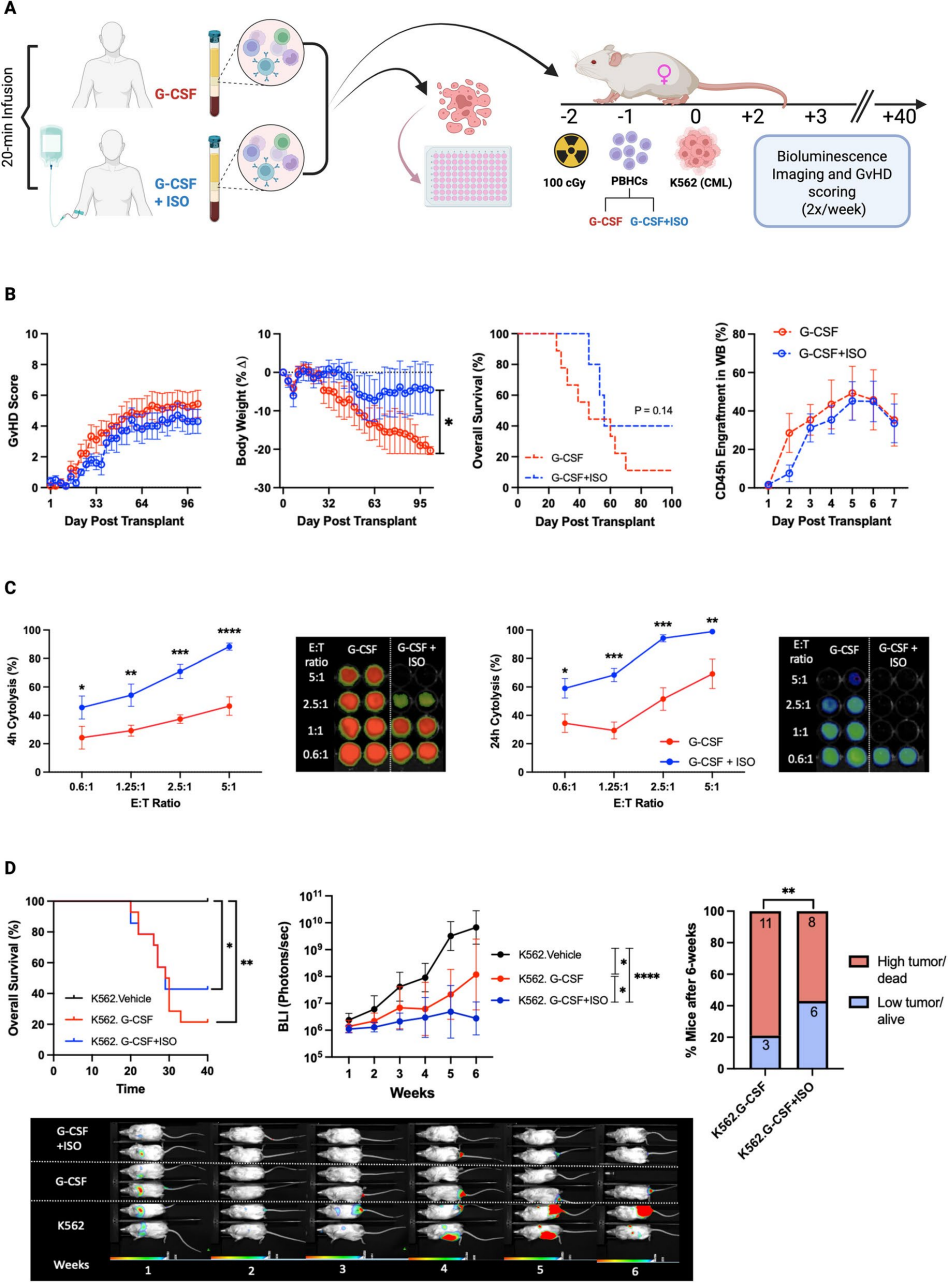
G-CSF + ISO Mobilized Grafts Enhance Survival, Reduce GvHD, and Improve Tumor Control in Xenogeneic Mice. Grafts collected after G-CSF or G-CSF + ISO mobilization were co-cultured with K562-luc cells to assess in vitro cytotoxicity and injected into xenogeneic mice to evaluate their graft-versus-host disease (GvHD) and graft-versus-leukemia (GvL) responses. (**A**) Schematic illustration of the experimental design. (**B**) NSG mice were injected with PBHCs (10 × 10⁶) collected after either G-CSF only (dashed red line) or G-CSF + ISO (dashed blue line) mobilization and monitored for GvHD score, body weight, survival, and engraftment. (**C**) PBHCs mobilized with G-CSF alone (red) or G-CSF + ISO (blue) were co-cultured with luciferase-tagged K562 cells at varying effector-to-target (E: T) ratios for 24 h. Data were collected at 4 and 24 h post-culture. (**D**) NSG-15 mice were injected with PBHCs (5 × 10⁶) collected after either G-CSF (red) or G-CSF + ISO (blue) mobilization and challenged with K562-luc (5 × 10⁵) (black). Mice were monitored for survival and tumor growth. Data are presented as mean ± SEM; *N* = 14. Statistical significance: *P ≤ 0.05, **P ≤ 0.01, ***P ≤ 0.001, ****P ≤ 0.0001

GvHD presents a major challenge in allogeneic hematopoietic stem cell transplantation (alloHSCT), often contributing to significant morbidity and increased mortality. To investigate the GvHD characteristics of grafts mobilized with G-CSF and ISO, xenogeneic mice (NSG) were transplanted with PBHCs mobilized using either G-CSF alone or G-CSF + ISO, and monitored for 100 days (Fig. 4A). While no statistically significant differences were observed in GvHD scores, mice receiving G-CSF mobilized PBHCs exhibited a significant decline in body weight throughout the experiment, whereas those receiving G-CSF + ISO mobilized PBHCs maintained their body weight (Fig. 4B). This is particularly significant as low body mass index (BMI) has been identified as an independent risk factor for mortality in patients with chronic GvHD [19]. Overall survival using Kaplan-Meier analysis revealed a trend (*p* = 0.14), with 40% of the mice injected with G-CSF + ISO PBHCs remaining alive by day 100 and exhibiting minimal signs of GvHD, compared to only 10% survival in the G-CSF alone group (Fig. 4B). This effect size was moderate (Cohen’s d = 0.66; 95% CI: 0.29 to 2.37), indicating a potentially meaningful clinical difference that may warrant further investigation in a larger cohort. No difference in engraftment was observed between the groups (*p* >0.05). These findings suggest that ISO + G-CSF-mobilized PBHCs mitigate GvHD and enhance survival outcomes in NSG mice.

### ISO infusion post-G-CSF enhances in vitro cytotoxicity and augments GvL effects in xenogeneic mice

A reduction in GvHD is often associated with a diminished ability of the graft to recognize and eliminate tumor cells. To evaluate in-vitro cytotoxicity, PBHCs mobilized with either G-CSF alone or G-CSF + ISO were co-cultured with luciferase-tagged K562 cells —a chronic myeloid leukemia cell line sensitive to NK cell cytolysis—at varying effector-to-target (E: T) ratios for 24 h. Data were collected at 4- and 24-hours post-culture. Across all E: T ratios and time points, PBHCs collected after G-CSF + ISO mobilization exhibited at least a 20% increase in cytotoxicity compared to PBHCs mobilized with G-CSF alone for each donor (Fig. 4C).

To validate the enhanced killing capacity in an in-vivo model, NSG-IL15 mice were injected with either G-CSF or G-CSF + ISO mobilized PBHCs and subsequently challenged with luciferase-tagged K562 cells. Although no difference in overall survival was noted between the experimental groups, both showed statistical significance from the vehicle control, suggesting mortality primarily resulted from GvHD rather than tumor progression. Mice receiving G-CSF + ISO mobilized grafts exhibited reduced bioluminescence imaging (BLI) signal intensity compared to those receiving G-CSF alone, suggesting enhanced tumor control. Additionally, by day 40, 42% of mice receiving G-CSF + ISO mobilized grafts were alive with low tumor burden, compared to only 21% in the G-CSF group (Fig. 4D). These findings indicate that ISO infusion enhances the GvL potential of G-CSF-mobilized PBHCs.

## Discussion

Enhancing the yield of CD34 + hematopoietic stem cells (HSCs) from allogeneic donors while favorably modifying the immune cell composition of grafts—without the need for ex vivo cell depletion or manipulation—remains a critical unmet need in alloHCT. To our knowledge, this is the first study to examine the use of a beta-adrenergic agonist (isoproterenol; ISO) to modulate the composition of G-CSF-mobilized PBHCs, with the potential to improve transplantation outcomes. We observed that ISO infusion following G-CSF mobilization modestly increased the concentration of CD34 + HSCs in the bloodstream while profoundly altering the immune cell composition and functional properties of the grafts. These G-CSF + ISO mobilized grafts exhibited distinct gene expression profiles and enriched pathways associated with anti-tumor activity, which correlated with enhanced in vitro cytotoxicity against the K562 chronic myeloid leukemia cell line. Furthermore, G-CSF + ISO mobilized PBHCs mitigated GvHD, improved survival, and significantly reduced K562 leukemic burden in xenogeneic mouse models.

In clinical practice, ongoing efforts focus on optimizing peripheral blood allogeneic hematopoietic cell grafts to improve outcomes after allogeneic hematopoietic cell transplantation (alloHCT). Key objectives include achieving successful engraftment, facilitating rapid immune reconstitution, enhancing graft-versus-leukemia (GvL) effects, and minimizing the risk of severe graft-versus-host-disease (GvHD) [20]. For more than three decades, G-CSF has been the primary method for mobilizing HSCs in both allogeneic donors and cancer patients, increasing peripheral blood CD34 + cell concentrations by 10–20-fold [21]. Adrenergic activation has also been explored as an alternative to G-CSF for mobilizing CD34 + HSCs, utilizing methods such as acute exercise, psychological stress, and ISO infusion [16, 17, 22, 23]. However, these approaches have not achieved the requisite CD34 + HSC concentrations in peripheral blood (>10 cells/µL) needed for transplantation [24]. While we have demonstrated that exercise can mobilize CD34 + HSCs via β2-AR -mediated mechanisms [16], a previous study found that ISO infusion alone failed to mobilize CD34 + HSCs in healthy volunteers [22]. Consistent with these findings, we observed here that ISO infusion prior to G-CSF mobilization did not significantly impact CD34 + HSC counts. However, when administered following G-CSF mobilization, ISO increased CD34 + cell numbers by ~ 29%, suggesting potential synergistic effects between G-CSF and adrenergic activation for mobilizing HSCs [25].

Although the increases in CD34 + cells with ISO infusion after G-CSF mobilization were modest, the true benefit of ISO lies in its profound effects on the immune cell composition of the graft, which are critical determinants of alloHCT outcomes [26]. Chang et al. reported that high numbers of naïve CD4 + T cells increased the incidence of grade II-IV acute GvHD (aGvHD) in patients with hematologic malignancies undergoing haploidentical alloHCT [27]. Meanwhile, Nachbaur et al. found that higher NK cell numbers in alloHCT grafts from HLA-identical siblings significantly reduced relapse risk [28]. Additionally, patients receiving a higher dose of CD56dim + NK cells from G-CSF-mobilized grafts had a lower risk of grade III-IV aGvHD and increased leukemia-free survival rates [29]. Here, we demonstrate that G-CSF + ISO mobilization enriches the CD34 + population along with NK cells and TCR-γδ T cells, while reducing the numbers of CD4 + T cells and B cells. Furthermore, Saliba et al. has shown that a higher CD4/CD8 ratio in the graft, is associated with a higher incidence of severe aGvHD [30]. In contrast, a higher prevalence of CD8 + T cells correlates with increased non-relapse mortality. Optimal transplant outcomes are typically associated with a CD4/CD8 ratio closer to 1 [30]. Our research found that ISO infusion following G-CSF mobilization resulted in a ~ 20% decrease in the CD4/CD8 ratio, indicating a shift towards a more balanced graft composition. The application of ISO infusion also extends to the post-transplant setting, where the G-CSF–mobilized graft can serve as a source for a manipulated donor lymphocyte infusion (DLI) product, or where additional lymphocytes can be collected from the donor for use as an unmanipulated DLI (i.e. non-G-CSF mobilized), with the goal of preventing or treating leukemic relapse [31]. Taken together, these findings suggest that ISO infusion optimizes graft composition, promoting a balance that may lead to better transplantation outcomes by enhancing GvL effects while mitigating GvHD risk.

In xenogeneic mice receiving G-CSF + ISO mobilized PBHCs, we observed a significant preservation of body weight compared to those receiving PBHCs mobilized with G-CSF alone, despite no statistical difference in GvHD scores. Importantly, by day 100, mice injected with G-CSF + ISO mobilized PBHCs showed a four-fold increase in survival compared to the G-CSF group, indicating a potential protective effect against GvHD and improved overall survival outcomes. NK cells, which are among the first immune cells to reconstitute following alloHCT, play a pivotal role against infections and facilitating anti-leukemia responses. Delayed recovery of mature NK cells can impair GvL effects and protection against infections [32]. Recently, NK cells have been implicated in modulating GvHD. Rivas and colleagues demonstrated in a murine model that NK cells can control the proliferation of antigen-activated CD4 + T cells, leading to reduced chronic GvHD, potentially through NKG2D receptor-ligand interactions [33]. Our results showed a significant increase in NKG2D + NK cells in the G-CSF + ISO mobilized grafts (Supplementary Fig. 1), which may partially explain the reduced GvHD observed in our xenogeneic model. However, further studies are needed to fully elucidate this potential mechanism.

Single-cell RNA sequencing revealed that G-CSF + ISO-mobilized PBHCs exhibited an enrichment of genes associated with cytotoxicity and anti-tumor activity. Key genes involved in cytotoxicity, such as *GZMB*,* GNLY*,* and PRF1*, along with the immune response gene *NKG7*, were upregulated in cytotoxic lymphocytes, including NK cells, TCR-γδ T cells, and CD8 + T cells. Notably, *NKG7* expression on NK cells has been demonstrated to be crucial in controlling cancer initiation, growth, and metastasis [34]. Additionally, *NKG7* was linked to the ability of CD8 + T cells and NK cells to translocate CD107a to the cell surface. CD107a is required for the release of perforin and granzyme B into target cells, inducing cell death [35]. Additional research from the same group showed that *NKG7* was upregulated on intertumoral antigen-specific CD8 + T cells and NK cells and was essential for the infiltration and accumulation of T cells within the tumor microenvironment [36]. These findings suggest that ISO infusion enhances the anti-tumor potential of mobilized PBHCs at a molecular level. Moreover, we observed a consistent increase in cytotoxic activity in PBHCs mobilized with G-CSF + ISO compared to those mobilized with G-CSF alone. Our in vivo studies further supported these findings, demonstrating reduced leukemia burden in mice receiving G-CSF + ISO-mobilized grafts.

A potential limitation of ISO infusion in the alloHCT setting is the robust cardiovascular responses it elicits. As expected, ISO administration resulted in elevated cardiac output and systolic blood pressure, reaching levels typically observed during moderate to vigorous intensity exercise [37, 38]. The dose used in this study (50 ng/kg/min) was specifically chosen for its ability to preferentally mobilize NK-cells into the peripheral ciculation, analagous to vigorous exercise performed at 70% VO2max [38]. While these physiologically responses may cause discomfort in some individuals – paticularly in the absence of metabolic demand that normally accompanies exercise – this limitation could potentially be addressed by pre-treating donors with a selective β1AR-antagonist. Since approximately 75% of cardiac β-adrenergic receptors are of the β1 subtype, β1AR blockade would reduce ISO binding in the heart while enhancing its selective activation of β2ARs, which are primarily responsible for mobilizing effector lymphocytes during systemic adrenergic stimulation. Thus, co-administration of ISO with a β1AR antagonist such as bisoprolol may further enhance graft quality while attenuating cardiovascular side effects, as we have seen with vigorous exercise [38]. This approach could improve donor comfort during prolonged HSC collections, which often exceed three hours in duration.

Although we did not perform direct mechanistic experiments to link our current findings to β-adrenergic signaling, our most recent publication demonstrated that blocking β2-adrenergic receptor (β2-AR) signaling—either systemically or specifically—abolishes exercise-induced mobilization of lymphocytes, particularly NK cells, indicating this effect is β2-AR dependent [38]. We further found that the NK cells mobilized during exercise via β2-AR signaling were essential for tumor control: both β2-AR signaling and NK cells were required for voluntary wheel running to protect against murine B-cell lymphoma in immunocompetent mice [38]. Together, these findings provide strong mechanistic evidence that β2-AR engagement directly regulates both immune cell mobilization and anti-tumor function. However, our study is limited by a small sample size (*n* = 10), consisting primarily of young, healthy donors. Larger clinical trials with more diverse donor populations, including an apheresis procedure, are needed to validate these findings and assess the feasibility of incorporating ISO infusion into standard alloHCT protocols. Donor-specific variability in adrenergic response, sex-related differences, and potential hemodynamic effects also warrant further investigation, including the possible role of cardiac selective beta-blockers to manage these responses. Future studies using more advanced models, such as humanized NSG-HLA mice with defined HLA mismatches, will be critical to evaluate ISO’s effects in the context of alloreactive T-cell dynamics, immune reconstitution, and true GvHD development. Finally, mechanistic studies are needed to elucidate the pathways by which ISO modulates graft function and anti-tumor immunity.

## Conclusion

Infusing ISO during HSC collections after G-CSF mobilization favorably modulates graft composition by enriching beneficial cell populations such as CD34⁺ hematopoietic stem/progeneitor cells, NK cells, and TCR-γδ T cells, while reducing B cells and CD4⁺ T cells that are known to be associated with adverse outcomes such as GvHD. This optimized graft profile translated into enhanced cytotoxic activity in vitro, and, in xenogeneic mouse models, reduced GvHD severity, improved tumor control, and extended survival. These findings highlight the potential of ISO as a novel adjunct to G-CSF mobilization strateges, not only to improve HSC graft quality but also to augment donor lymphocyte infusion products, ultimately supporting better clinical outcomes following alloHCT.

## Supplementary Information

Below is the link to the electronic supplementary material.

Supplementary Material 1.

Supplementary Material 2.

## Data Availability

The sequencing data generated and analyzed in this study have been deposited in the Gene Expression Omnibus (GEO) repository under accession number GSE309253. Additional data are available from the corresponding author upon request.
