# Supplementary material for: Isoproterenol infusion enhances composition and function of G-CSF mobilized allogeneic peripheral blood hematopoietic cell grafts
Source: Stem Cell Res Ther. 2025 Nov 5;16:614. doi: 10.1186/s13287-025-04725-4 (PMC12587703; doi:10.1186/s13287-025-04725-4)
Supplement: Supplementary file 2 — Supplementary Material 2. [file 13287_2025_4725_MOESM2_ESM.pdf]

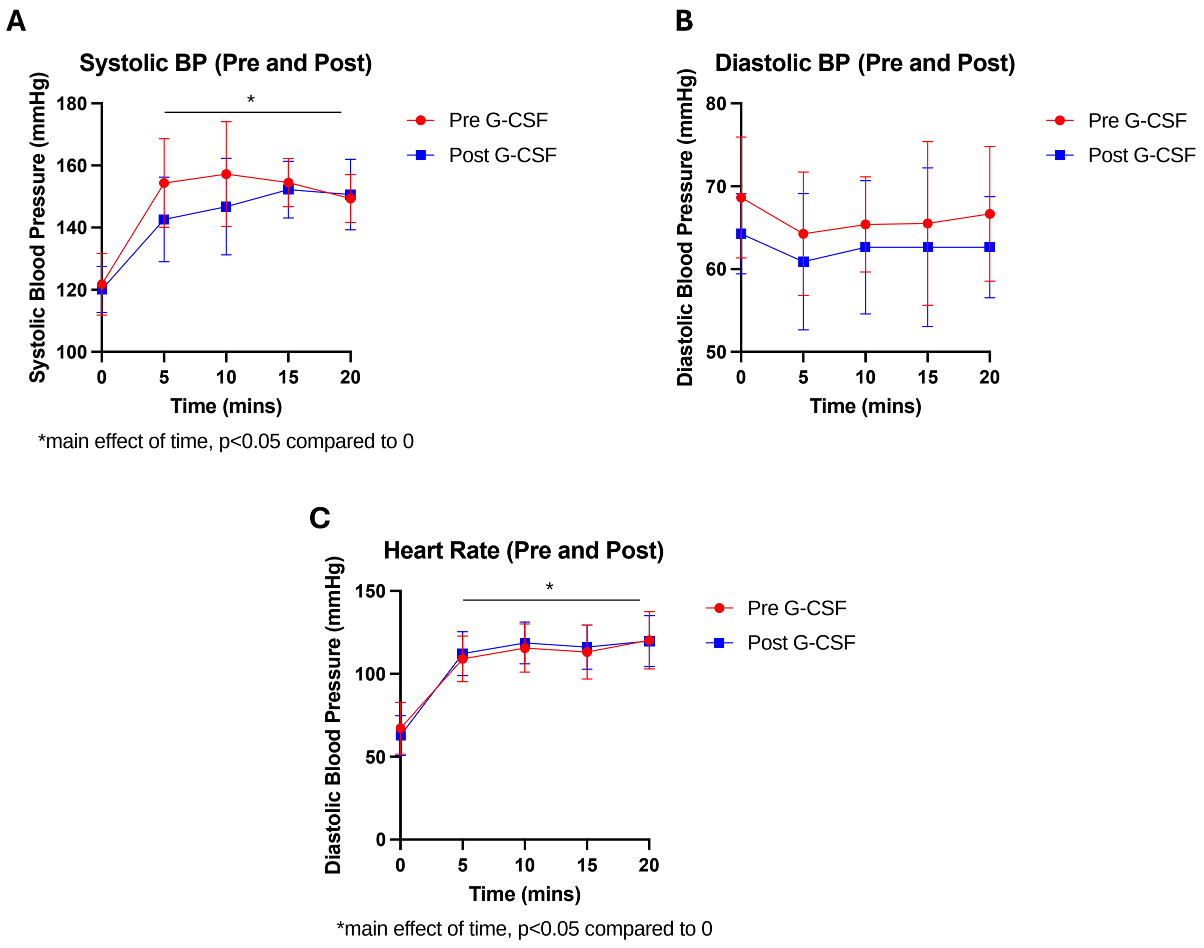

Supplemental Figure 1 – ISO Infusion Increases Systolic Blood Pressure and Heart Rate.

Systolic and diastolic blood pressure, along with heart rate, were measured every 5 minutes during ISO infusion. Regardless of prior G-CSF mobilization, ISO infusion consistently elevated systolic blood pressure and heart rate, with no significant differences between trials.

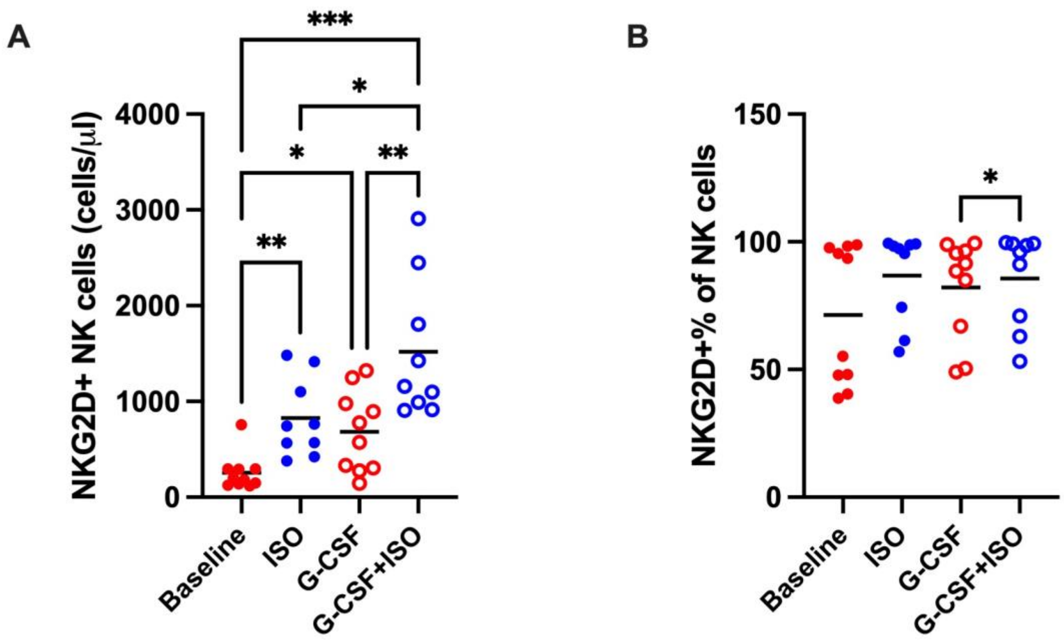

Supplemental Figure 2 – ISO Infusion Preferentially Mobilizes NKG2D+ NK Cells. Total cell count (cells/μL) (A) and percentage (B) of NK cells expressing the activating receptor NKG2D were assessed at baseline (solid red), after ISO infusion (solid blue), following G-CSF treatment (open red), and after combined G-CSF+ISO treatment (open blue). Data are presented as mean ± SEM; N = 10. Statistical significance: *P ≤ 0.05, **P ≤ 0.01, ***P ≤ 0.001.

Supplemental Table 1 – Antibody panels

	VioBlue	VioGreen	FITC	PE	PerCP-Vio700	PE-Vio770	APC	APC-Vio770
	450/50 nm	525/50 nm	525/50 nm	585/40 nm	655–730 nm	750 nm LP	655–730 nm	750 nm LP
**Panel 1**	CD8	CD14	CD3	CD4	CD20		CD45	CD56
**Panel 2**	CD8	CD3	CD4	CD62L		CD45RA	CD45	CD56
**Panel 4**		CD3		NKG2D	PI		CD45	CD56
**Panel 4**			CD34	CD133	PI		CD45	
